# Incorporation of kartogenin and silk fibroin scaffolds promotes rat articular cartilage regeneration through enhancement of antioxidant functions

**DOI:** 10.1093/rb/rbad074

**Published:** 2023-08-31

**Authors:** Zejun Pan, Mingzhuang Hou, Yijian Zhang, Yang Liu, Xin Tian, Xiayu Hu, Xiaoyang Ge, Zhijian Zhao, Tao Liu, Yong Xu, Huilin Yang, Hao Liu, Xuesong Zhu, Fan He

**Affiliations:** Department of Orthopaedics, The First Affiliated Hospital of Soochow University, Soochow University, Suzhou 215006, China; Orthopaedic Institute, Suzhou Medical College of Soochow University, Soochow University, Suzhou 215007, China; Department of Orthopaedics, People's Hospital of Zhenhai District (Ningbo No.7 Hospital), Ningbo 315202, China; Department of Orthopaedics, The First Affiliated Hospital of Soochow University, Soochow University, Suzhou 215006, China; Orthopaedic Institute, Suzhou Medical College of Soochow University, Soochow University, Suzhou 215007, China; Department of Orthopaedics, The First Affiliated Hospital of Soochow University, Soochow University, Suzhou 215006, China; Orthopaedic Institute, Suzhou Medical College of Soochow University, Soochow University, Suzhou 215007, China; Department of Orthopaedics, The First Affiliated Hospital of Soochow University, Soochow University, Suzhou 215006, China; Orthopaedic Institute, Suzhou Medical College of Soochow University, Soochow University, Suzhou 215007, China; Department of Orthopaedics, The First Affiliated Hospital of Soochow University, Soochow University, Suzhou 215006, China; Orthopaedic Institute, Suzhou Medical College of Soochow University, Soochow University, Suzhou 215007, China; Department of Orthopaedics, The First Affiliated Hospital of Soochow University, Soochow University, Suzhou 215006, China; Orthopaedic Institute, Suzhou Medical College of Soochow University, Soochow University, Suzhou 215007, China; Department of Orthopaedics, The First Affiliated Hospital of Soochow University, Soochow University, Suzhou 215006, China; Orthopaedic Institute, Suzhou Medical College of Soochow University, Soochow University, Suzhou 215007, China; Department of Orthopaedics, The First Affiliated Hospital of Soochow University, Soochow University, Suzhou 215006, China; Orthopaedic Institute, Suzhou Medical College of Soochow University, Soochow University, Suzhou 215007, China; Department of Orthopaedics, The First Affiliated Hospital of Soochow University, Soochow University, Suzhou 215006, China; Orthopaedic Institute, Suzhou Medical College of Soochow University, Soochow University, Suzhou 215007, China; Department of Orthopaedics, The First Affiliated Hospital of Soochow University, Soochow University, Suzhou 215006, China; Orthopaedic Institute, Suzhou Medical College of Soochow University, Soochow University, Suzhou 215007, China; Department of Orthopaedics, The First Affiliated Hospital of Soochow University, Soochow University, Suzhou 215006, China; Orthopaedic Institute, Suzhou Medical College of Soochow University, Soochow University, Suzhou 215007, China; Department of Orthopaedics, The First Affiliated Hospital of Soochow University, Soochow University, Suzhou 215006, China; Department of Orthopaedics, The First Affiliated Hospital of Soochow University, Soochow University, Suzhou 215006, China; Orthopaedic Institute, Suzhou Medical College of Soochow University, Soochow University, Suzhou 215007, China; Department of Orthopaedics, The First Affiliated Hospital of Soochow University, Soochow University, Suzhou 215006, China; Orthopaedic Institute, Suzhou Medical College of Soochow University, Soochow University, Suzhou 215007, China

**Keywords:** articular cartilage, silk fibroin, kartogenin, extracellular matrix, redox balance, NRF2

## Abstract

Treating articular cartilage defects in patients remains a challenging task due to the absence of blood vessels within the cartilage tissue. The regenerative potential is further compromised by an imbalance between anabolism and catabolism, induced by elevated levels of reactive oxygen species. However, the advent of tissue engineering introduces a promising strategy for cartilage regeneration, offering viable solutions such as mechanical support and controlled release of chondrogenic molecules or cytokines. In this study, we developed an antioxidant scaffold by incorporating natural silk fibroin (SF) and kartogenin (KGN)-loaded liposomes (SF-Lipo@KGN). The scaffold demonstrated appropriate pore size, connectivity, and water absorption and the sustained release of KGN was achieved through the encapsulation of liposomes. *In vitro* experiments revealed that the SF-Lipo@KGN scaffolds exhibited excellent biocompatibility, as evidenced by enhanced cell adhesion, migration, and proliferation of chondrocytes. The SF-Lipo@KGN scaffolds were found to stimulate cartilage matrix synthesis through the activation of the nuclear factor erythroid-2-related factor 2/heme oxygenase-1 antioxidant signaling pathway. *In vivo* experiments demonstrated the effective promotion of articular cartilage regeneration by the SF-Lipo@KGN scaffolds, which enhanced extracellular matrix anabolism and restored the intrinsic redox homeostasis. Overall, this study successfully developed biomimetic KGN-loaded scaffolds that restore cartilage redox homeostasis, indicating promising prospects for cartilage tissue engineering.

## Graphical Abstract

**Figure:**
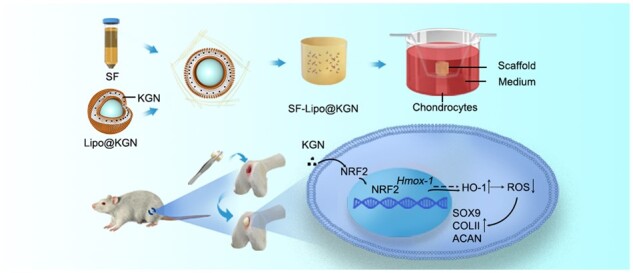

## Introduction

Articular cartilage, a transparent connective tissue that envelops the surfaces of articulating bones, serves to minimize friction between adjacent bones [1]. The occurrence of articular cartilage defects is a widespread pathology associated with osteoarthritis, and is a major cause of global disability [2]. This condition is typically attributed to repetitive mechanical loading, inflammation, and acute injury [3]. The regenerative capacity of articular cartilage is limited due to the absence of blood vessels, nerves, and lymphatics [4]. Despite the availability of several treatments for cartilage defects, such as micro-drilling, microfracture surgery, and autologous tissue transplantation, none of these approaches are capable of regenerating hyaline cartilage [5]. Consequently, there is a pressing need for innovative tissue engineering strategies to expedite the repair and regeneration of cartilage.

Silk fibroin (SF), a naturally occurring protein, has been extensively utilized in various forms within the realm of tissue engineering [6, 7]. The adjustable biomechanical characteristics of SF scaffolds can effectively facilitate reciprocal interactions between biomaterials and adjacent cells [8, 9]. The mechanical, swelling, and degradation properties of the silk II (β-sheet) content in the SF scaffolds varied depending on the modification time, indicating a notable level of plasticity [10]. In comparison to other natural biomaterials like collagen, SF demonstrates superior mechanical properties *in vivo*. Additionally, SF scaffolds create a favorable milieu for cell proliferation, differentiation, and matrix remodeling [11]. Prior research has demonstrated that SF scaffolds enhance the expression of cartilage matrix components, underscoring its potential for hyaline cartilage repair [12, 13]. However, the utilization of SF scaffolds in cartilage tissue engineering presents a formidable obstacle.

Hyaline cartilage, characterized by its intricate organization and absence of blood vessels, possesses limited regenerative capabilities due to its sparse cell population and inadequate blood supply [14]. SF scaffolds are unable to completely replicate the intricate microenvironment of cartilage, which encompasses cellular interactions with growth factors, cytokines, and other constituents of the cartilage extracellular matrix (ECM) [15]. In order to surmount these aforementioned limitations, the incorporation of bioactive molecules into the scaffolds becomes imperative to augment chondrocyte proliferation and cartilage matrix synthesis.

Over the past decade, there has been extensive research on the potential of kartogenin (KGN) in promoting the regeneration of cartilage, bone–tendon junctions, and discs [16]. KGN has demonstrated the ability to enhance the proliferation and chondrogenesis of bone marrow-derived mesenchymal stem cells [17]. Notably, in a rabbit model with articular cartilage defects, the application of KGN-coated hydrogel resulted in superior cartilage repair compared to untreated controls [18]. Furthermore, in a mouse osteoarthritis model, intra-articular injection of KGN effectively mitigated cartilage erosion and reduced inflammation in the joints [19]. Furthermore, previous studies have demonstrated that KGN possesses anti-inflammatory properties, effectively inhibiting the production of pro-inflammatory cytokines and chemokines induced by lipopolysaccharide in both human synovial fibroblasts and chondrocytes. This suggests that KGN exhibits a dual effect of promoting regeneration while also reducing inflammation [20]. However, the direct administration of KGN faces limitations in clinical settings due to its short half-life, hydrophobic nature, and potential adverse reactions in non-targeted tissues [21]. To address these challenges, the utilization of nano-scaled phospholipid bilayer membrane-structured liposomes has emerged as a promising approach for encapsulating KGN. These liposomes have been extensively employed for the delivery of small molecule drugs, making them a suitable vehicle for the controlled release of KGN [22]. Therefore, the utilization of liposomes for the delivery of KGN has the potential to augment the pharmacodynamics of retention.

The alteration of the microenvironment subsequent to injury plays a pivotal role in determining the efficacy of cartilage regeneration. Anomalous oxidative stress, encompassing free radicals, peroxides, and superoxide anions, detrimentally impacts chondrocyte fate and hinders the reparative mechanisms [23]. Reactive oxygen species (ROS) are a collection of exceedingly reactive molecules that serve as secondary messengers in numerous biological processes, such as cellular proliferation, differentiation, and apoptosis. Although the antioxidant defense system present in healthy cartilage tissues works to counteract ROS, an excessive production hinders the synthesis of cartilage matrix and promotes its degradation. ROS can directly compromise the structural components of cartilage ECM, including collagen and proteoglycans, resulting in their degradation and subsequent loss of mechanical properties [24]. Moreover, over-production of ROS induces cartilage ECM degradation by activating the nuclear factor-kappa B pathway (NF-κB)-related matrix metalloproteinases (MMPs) [25], and causes chondrocyte apoptosis through the initiation of the caspase cascade pathway [26]. However, recent findings have demonstrated that KGN can mitigate the detrimental effects of ROS by activating the nuclear factor erythroid-2-related factor 2 (NRF2) antioxidant network [19]. It has been established that the coordination of intrinsic redox balance plays a crucial role in maintaining mitochondrial homeostasis and augmenting chondrocyte anabolism [27]. Therefore, the development of biomaterials that can adapt to the microenvironment and regulate the balance between oxidants and antioxidants *in vivo* is advantageous for the remodeling of cartilage matrix.

Inspired by the redox microenvironment observed during the cartilage remodeling process, we have designed a composite scaffold consisting of SF and liposome-encapsuled KGN. This scaffold not only provides mechanical support for cartilage repair, but also releases KGN in a sustained manner to modulate redox equilibrium and promote anabolic metabolism of cartilage ECM. We have conducted *in vitro* and *in vivo* assessments to evaluate the impact of the SF-Lipo@KGN scaffolds on ECM synthesis. We have also investigated the underlying mechanisms by which the SF-Lipo@KGN scaffolds regulated the redox homeostasis, specifically the intracellular antioxidant signaling pathways.

## Materials and methods

### Preparation of SF solution

SF solution was prepared as previously reported [28]. The silk (Bombyx mori, Zhejiang Xingyue Biotechnology, Hangzhou, China) was immersed in 0.02 M sodium carbonate (Aladdin, Shanghai, China) aqueous solution and boiled at 100°C for 90 min. The sericin was then washed away with distilled water. The purified silk fibers were dissolved in 9.3 M lithium bromide (LiBr, Aladdin) at 60°C for 6 h. The solution was transferred to the cellulose membrane (molecular weight 3500 MW) and dialyzed in distilled water to remove impurities for 72 h. Subsequently, the SF solution was concentrated to 100 mg/ml and stored at 4°C.

### Fabrication of SF-Lipo@KGN scaffolds

Liposomes were prepared using the thin-film disperse method as previously reported [29]. Briefly, 40 mg of lecithin, 10 mg of cholesterol, 2 mg of octadecylamine (all from Aladdin), and 5 mg of KGN (Sigma-Aldrich, St. Louis, MO, USA) were dissolved in 5 ml of chloroform (Sigma-Aldrich). The organic solvent was removed through rotary evaporation at 37°C for 2 h. The lipid film was hydrated with deionized water and sonicated at 37°C until completely dissolved. Then 2% mannitol (Aladdin) was added to the mixture of SF solution and liposome solution (1:1), which acted as a lyoprotectant to maintain the integrity of liposomes during freeze-drying and rehydration. After injected the mixture into a silicone mold (1.5 mm in diameter and 1.5 mm in height), the mixture was freeze-dried for 72 h.

### Characterization of liposomes encapsuled KGN

The diameter and Zeta potential of liposomes were measured with dynamic light scattering (Zetasizer Nano S, Malvern, UK). The morphology of liposomes was observed with transmission electron microscope (TEM, Tecnai G2 Spirit Biotwin, FEI, Hillsboro, OR, USA). Before observation under TEM, the liposome solution was negatively stained with 5% phosphotungstic acid (Sigma-Aldrich). KGN was quantified by reverse-phase HPLC (HPLC-1260, Agilent, Infinite, Germany). The analysis was carried out with a flow rate of 0.5 ml/min and recorded at 274 nm for 10 min. The content of KGN was determined based on the calibration curve.

### Scanning electron microscopy

The surface of the scaffolds was sprayed with Au layer by ion sputtering instrument (Quanta 250, FEI). The morphological characteristics of the scaffolds were analyzed by scanning electron microscopy (SEM) (NovaNano 450, FEI) at voltage of 10 kV.

### Swelling ratio

The dry weight of each scaffold was estimated as *W*_0_ and then scaffolds were immersed in 5 ml of phosphate-buffered saline (PBS, Thermo Fisher Scientific, Waltham, MA, USA) at 37°C. The scaffolds were taken out of PBS at several predefined times, and their wet weights were recorded as *W*_t_. The swelling rate is determined by the following formula: swelling ratio = (*W*_t_ − *W*_0_)/*W*_0_ × 100%.

### Degradation rate

The dry scaffolds were weighed as *M*_0_ and then immersed in PBS for 4 weeks at 37°C. At predefined time points, the weight of scaffolds was weighed and recorded as *M*_t_ after drying at 60°C for 4 h. Degradation rate was calculated as follows: remaining weight=*M*_t_/*M*_0_ × 100%.

### Release profile of KGN

The scaffolds were immersed in PBS at 37°C. All supernatants were absorbed and the initial volume of PBS was supplemented at different predefined time points for 8 weeks. The concentration of KGN in the supernatant was measured by HPLC.

### Mechanical evaluation

The compressive properties of scaffolds were measured using a mechanical testing machine (Hengyi Co., Ltd, Shanghai, China) at a compressive speed of 0.5 mm/min until the scaffold was broken. The compressive modulus was determined using the stress–strain curve's slope.

### Isolation and culture of articular chondrocytes

All animal experiments were approved by the Ethics Committee of Soochow University (SUDA20220926A05). Articular cartilage tissue was extracted from knee joint of Sprague-Dawley (SD) rats (male, 8 weeks old). After scraping off the tissues with a sterile blade, cartilage was minced into little pieces (1 mm^3^) using a microscissor. Cartilage fragments were digested in Dulbecco's Modified Eagle Medium/Nutrient Mixture F-12 (DMEM/F12) medium containing 2 mg/ml type II collagenase (Thermo Fisher Scientific) for 6 h. After digesting for 6 h, the chondrocytes were centrifuged and cultured in DMEM/F12 supplemented with 10% fetal bovine serum (FBS) and 1% penicillin-streptomycin (Thermo Fisher Scientific). Chondrocytes at passage one were used for the following experiments.

### Cells proliferation

Cell Counting Kit-8 (CCK-8, Beyotime, Haimen, China) reagent was used to analyze chondrocyte proliferation. After culturing for 1, 3, 5, and 7 days, chondrocytes were incubated with CCK-8 working solution at 37°C for 1 h. The optical density (OD) at a wavelength of 450 nm was quantified with a multiplate reader (BioTek, Winooski, VT, USA).

### Cell viability

Briefly, 1 × 10^5^ chondrocytes were seeded into a 24-well plate and cultured with scaffolds. On days 1, 3, and 5, chondrocytes were incubated with the Live/Dead staining kit (Beyotime) for 30 min. Images of live and dead cells were taken by a fluorescence microscope (Zeiss, Oberkusen, Germany).

### Cell migration

Scratch wound assays were performed when the cell confluence reached 90%. After scraping with the tip of sterile pipette, the chondrocytes were cultured with migration medium (DEME/F12 containing 0.1% FBS) [30]. The scratch wounds were photographed using an optical microscope (Zeiss) at various time intervals.

### Cell tracing experiments

The scaffolds were pretreated with DEME/F12 for 6 h. 3,3′-Dioctadecyloxacarbocyanine perchlorate (DIO)-labeled chondrocytes (Beyotime) were seeded on the surfaces of scaffolds and protected from light. A fluorescence microscopy (Zeiss) was used to capture images of chondrocytes growth after cultures were maintained for 1, 3, and 5 days.

### Quantitative reverse transcription-polymerase chain reaction (qRT-PCR)

After 7 days of co-culture with different scaffolds, the total RNA of chondrocytes was extracted by TRIzol reagent (Thermo Fisher Scientific), and complementary DNA (cDNA) was prepared using the cDNA kit (Thermo Fisher Scientific). The reaction system of quantitative reverse transcription-polymerase chain reaction (qRT-PCR) was performed using the SYBR Green Master Supermix kit (Bio-Rad, Hercules, CA, USA). Gene expression was analyzed using qRT-PCR with CFX96TM real-time PCR instrument (Bio-Rad). Glyceraldehyde-3-phosphate dehydrogenase (*Gapdh*) was determined as an internal control and the relative expression of target gene was calculated with *χ* = 2^−ΔΔCT^ method. The primer sequences are listed in Table 1.

**Table 1.** Primers used for qRT-PCR

Gene	Forward primer sequence (5ʹ–3ʹ )	Reverse primer sequence (5ʹ–3ʹ)
*Gapdh*	GCAAGTTCAACGGCACAG	CGCCAGTAGACTCCACGAC
*Col2a1*	CCTGGACCCCGTGGCAGAGA	CAGCCATCTGGGCTGCAAAG
*Acan*	AGGATGGCTTCCACCAGTGC	TGCGTAAAAGACCTCACCCTCC
*Sox9*	TAAATTCCCAGTGTGCATCC	GCACCAGGGTCCAGTCATA
*Nrf2*	GCTATTTTCCATTCCCGAGTTAC	ATTGCTGTCCATCTCTGTCAG
*Sod2*	GGCCAAGGGAGATGTTACAA	GCTTGATAGCCTCCAGCAAC
*Ho-1*	CTTTCAGAAGGGTCAGGTGTC	TGCTTGTTTCGCTCTATCTCC

### Western blot

The protein of chondrocytes was isolated with radioimmunoprecipitation assay lysis buffer (RIPA, Beyotime). The concentration of protein was determined using the bicinchoninic acid (BCA) protein assay kit (Beyotime). The equal amount of protein extract was separated using 10% sodium dodecyl sulfate-polyacrylamide gel electrophoresis (SDS-PAGE, Beyotime) and transferred onto a nitrocellulose membrane (Beyotime). After blocked with blocking buffer (Beyotime), the membrane was incubated with different primary antibodies: anti-COL II (ab188570), anti-ACAN (ab36861), anti-SOX9 (ab185966), anti-GPX1 (ab108429), anti-SOD2 (ab13533), anti-heme oxygenase-1 (HO-1) (ab6994), anti-NRF2 (ab62352, all from Abcam, Cambridge, UK), and anti-α-Tubulin (Beyotime). The membranes were then incubated with secondary antibodies labeled with horseradish peroxidase (Abcam). The intensity of protein bands was visualized by enhanced chemiluminescence solution (Thermo Fisher Scientific) and quantified with ImageJ software (National Institutes of Health, Bethesda, MD, USA).

### Immunofluorescence staining

Chondrocytes in 24-well plates were fixed with 4% paraformaldehyde (Aladdin) and then penetrated with 0.1% Triton X-100 (Beyotime) for 10 min. After blocking for 30 min, the chondrocytes were incubated at 4°C for 8 h with anti-COL II (ab34712), anti-NRF2 (ab62352), and anti-SOD2 (ab13533, Abcam). Subsequently, the chondrocytes were incubated for 1 h with a secondary antibody (ab150075, Abcam) mixed with phalloidin (C1033, Beyotime). The nucleus was counterstained with 4',6-diamidino-2-phenylindole (DAPI, Beyotime). For ROS staining, the chondrocytes were incubated with 10 μM 2',7'−dichlorodihydrofluorescein diacetate (DCFH-DA, Beyotime) for 10 min at 37°C in the dark. Images were captured using a fluorescence microscope (Zeiss).

### Establishment of a rat cartilage defect model

Twenty-four SD male rats were obtained from the Animal Experimental Center of Soochow University and randomly distributed into four groups: the sham group, the defect group, the SF-Lipo group, and the SF-Lipo@KGN group. The rats were anesthetized with pentobarbital sodium (40 mg/kg, Chemical Reagent Company, Shanghai, China). After exposing the femoral condyle, full-thickness cartilage defects (1.5 mm in diameter and 1.5 mm in depth) were made at the trochlear groove with electric trephine. The scaffold was then implanted into the cartilage defect. The patellar ligament was restored and the wound was sewed layer by layer. To avoid infection, penicillin was injected into rat within 3 days after the surgery. After undergoing surgery for 6 and 12 weeks, the rats were sacrificed by an overdose of pentobarbital (150 mg/kg) and knee joint specimens were collected.

### Histology and immunohistochemistry

The samples were decalcified in 10% ethylene diamine tetra acetic acid (EDTA, Aladdin) for 4 weeks and then sectioned into 5-µm-thick sections. Before staining, the sections were deparaffinized with xylene for 20 min and dehydrated with gradient alcohol for 10 min each. The sections were stained with hematoxylin and eosin (H&E) and Safranin O (S.O.)/Fast Green (Sigma-Aldrich) according to the manufacturer’s instructions. Three independent observers scored cartilage using the International Cartilage Repair Society (ICRS) macroscopic score system and modified O'Driscoll grading system (MODS) [31].

For immunohistochemistry, the sections were blocked with 1% 1% bovine serum albumin (BSA, Aladdin) after incubating with 2 mg/ml hyaluronidase (Aladdin). The sections were incubated at 4°C with anti-COL II (ab34712), anti-SOD1 (ab13498), and anti-NRF2 (ab62352, Abcam) overnight. Subsequently, the sections were incubated with HRP-conjugated goat anti-rabbit IgG (ab6721, Abcam) for 1 h and visualized using 3,3′-diaminobenzidine solution (DAB, Sangon Biotech, Shanghai, China). The percentage of positive cells was quantitatively evaluated using ImageJ software.

### Statistical analysis

GraphPad Prism 9.2 software (GraphPad Software Inc., USA) was applied for statistical analysis. The data are displayed as mean ± standard deviation. The independent two-tailed Student's *t*-test was used to compare the data between two groups. Multiple group comparisons were conducted using a one-way analysis of variance (ANOVA) followed by Tukey's *post hoc* test. A value of *P* less than 0.05 (*) or 0.01 (**) was considered statistically significant.

## Results

### Fabrication and characterization of SF-Lipo@KGN scaffolds

**Figure 1.**
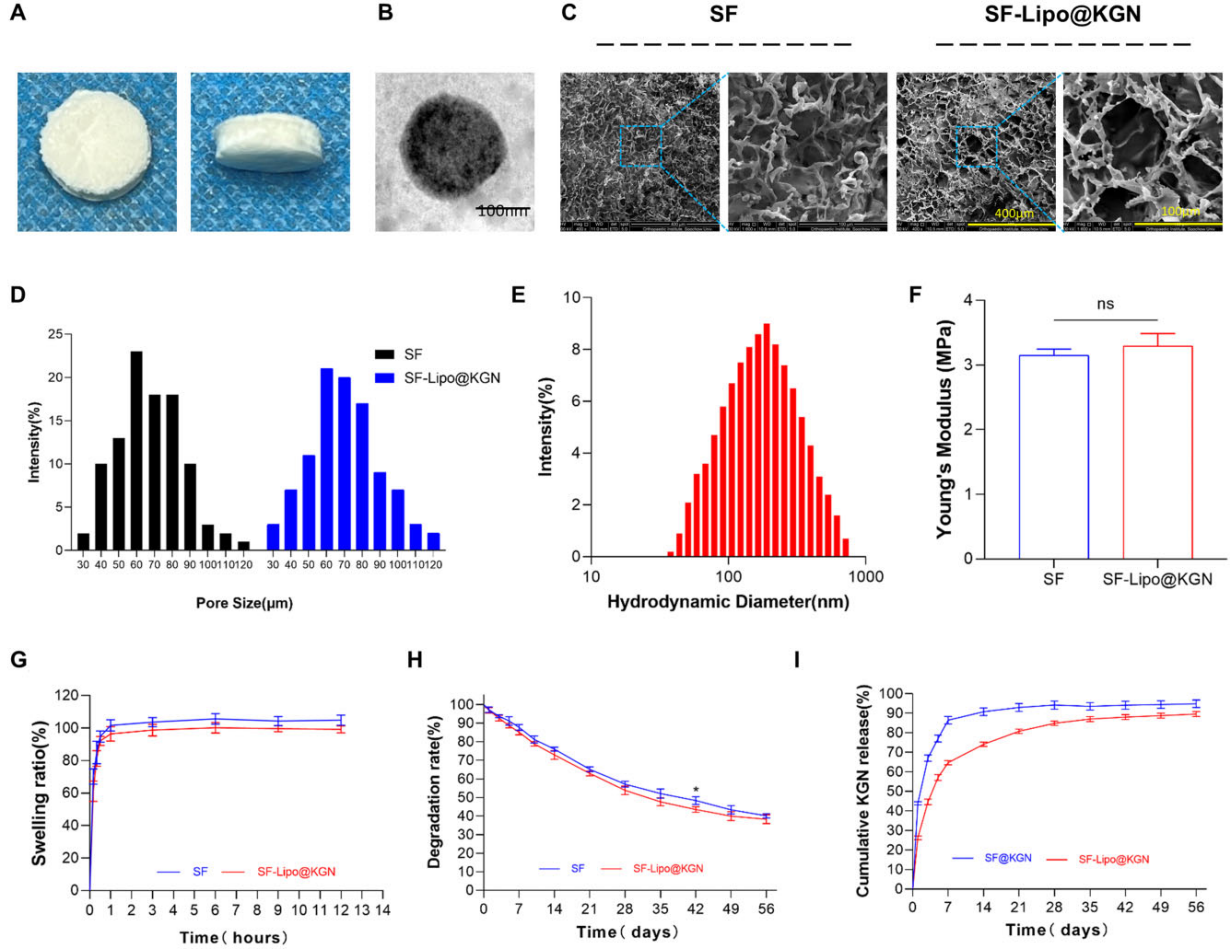
Characterization of SF and SF-Lipo@KGN scaffolds. (**A**) The general view of the SF-Lipo@KGN scaffold. (**B**) The TEM result of lipo@KGN. Scale bar = 100 nm. (**C, D**) SEM images and pore size distribution of the SF and SF-Lipo@KGN scaffold. Scale bar = 400 μm and 100 μm. (**E**) Hydrodynamic diameter distribution of liposomes measured by DLS. (**F–H**) Young’s modulus, swelling ratio, and degradation of SF and SF-Lipo@KGN scaffold. (I) KGN release behavior at each time point within 8 weeks. The data are presented as mean ± SD (*n* = 3). ns represents no significant difference. *Indicates statistically significant differences where *P *<* *0.05.

After undergoing the drying process, the concentration of SF solution was determined to be 5%. Subsequently, the solution was concentrated through dialysis, and then combined with an equal volume of KGN-loaded liposomes to produce SF-Lipo@KGN composite scaffolds (Figure 1A). The preparation of liposomes was carried out using the thin-film disperse method, with monodisperse liposomes being obtained through repeated sonication during rehydration (Figure 1B). The SEM images revealed that the interconnected porous structure exhibited favorable characteristics for cell adhesion, migration, and deposition of the cartilage matrix (Figure 1C). The average diameter of the interconnected chambers within SF scaffolds and SF-Lipo@KGN scaffolds was measured to be 66.3 ± 18.9 μm and 70.3 ± 19.8 μm, respectively (Figure 1D). This indicates that the addition of liposomes did not significantly affect the porous network structure of SF scaffolds, as the pore sizes remained similar. The encapsulation efficiency of KGN in liposomes was determined to be 68.9 ± 2.4%. The average Zeta potential of the liposomes was found to be 21.5 ± 4.8 mV. Furthermore, the average hydrodynamic diameter of the monodisperse liposomes was measured to be 207.3 nm, with the majority falling within the range of 100–500 nm (Figure 1E).

In the mechanical test, both SF and SF-Lipo@KGN scaffolds exhibited comparable compression curves. The compression test revealed that the Young's modulus of the two scaffolds were measured to be 3.15 ± 0.09 MPa and 3.29 ± 0.19 MPa, respectively, suggesting that the inclusion of liposomes did not significantly affect the mechanical properties of the scaffolds (Figure 1F). Additionally, the scaffolds achieved swelling equilibrium within approximately 1 h with the swelling rate of SF scaffolds reaching 101.7 ± 3.4% and the swelling rate of SF-Lipo@KGN scaffolds reaching 96.5 ± 4.5% (Figure 1G). The scaffolds exhibited a commendable water absorption capacity, enabling them to retain bodily fluids and deliver nourishment to the cells. Following an 8-week degradation period *in vitro*, the SF and SF-Lipo@KGN scaffolds demonstrated degradation rates of 59.9 ± 1.2% and 61.7 ± 2.4%, respectively (Figure 1H). The release profile analysis revealed that liposomes played a crucial role in facilitating the sustained release of KGN. Specifically, the KGN released from SF@KGN scaffolds was barely detectable after 7 days, whereas SF-Lipo@KGN scaffolds exhibited a prolonged release of KGN for a duration of 5 weeks (Figure 1I).

### *In vitro* biocompatibility assessment of SF-Lipo@KGN scaffolds

**Figure 2.**
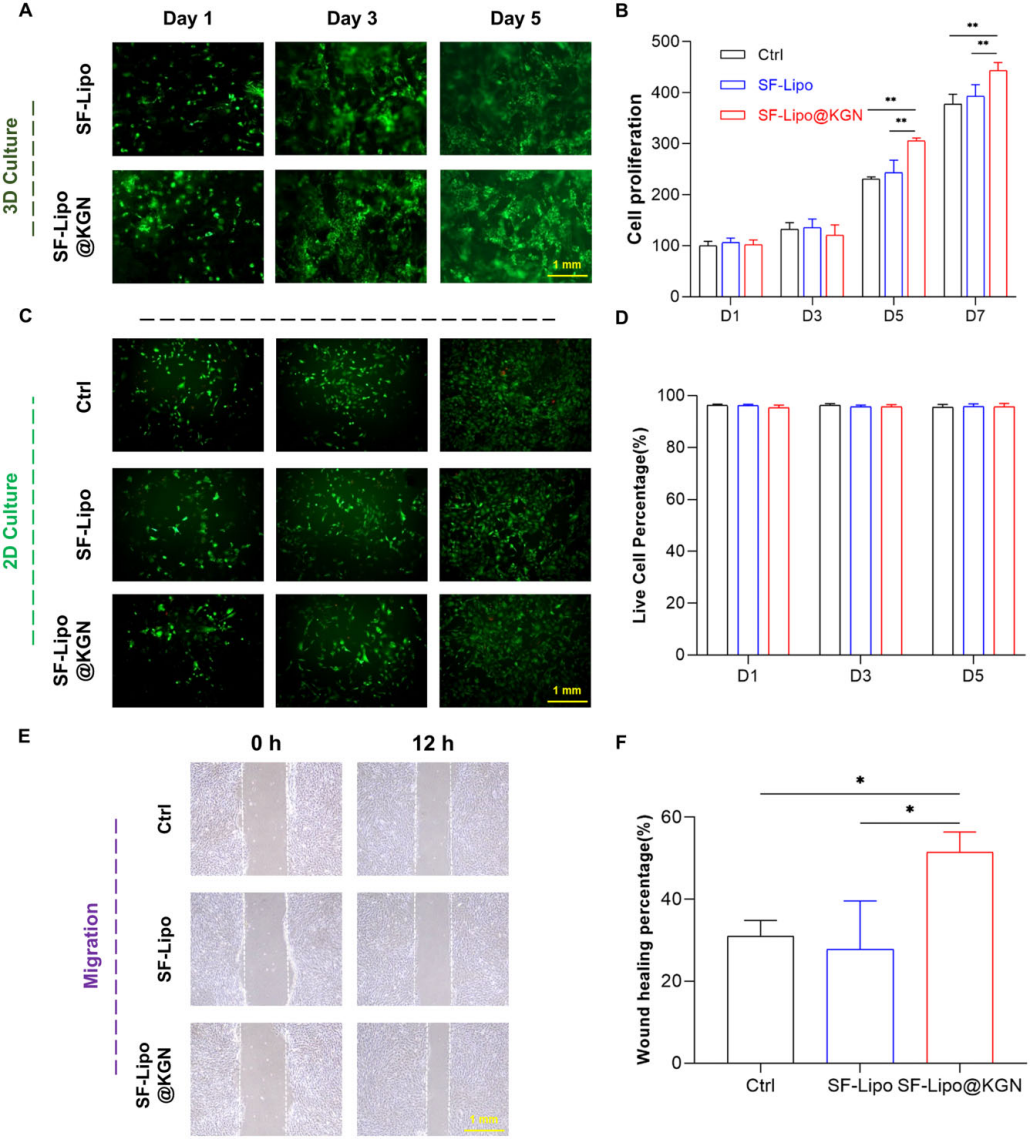
SF-Lipo@KGN scaffolds exhibited excellent biocompatibility. (**A**) Fluorescence image of DIO-labeled chondrocytes growing on a 3D scaffold. Scale bar = 1 mm. (**B**) CCK-8 was used to measure the effect of scaffolds on cell proliferation. (**C, D**) The survival of cells co-cultured with scaffold was determined using the live/dead staining. Scale bar = 1 mm. (**E, F**) Wound healing experiment to verify the effect of KGN on chondrocyte migration. Scale bar = 1 mm. The data are presented as mean ± SD (*n* = 3). *Indicates statistically significant differences where *P *<* *0.05, **where *P *<* *0.01.

To investigate the biological impact of composite SF-Lipo@KGN scaffolds on cell behavior, chondrocytes were labeled with DIO and introduced onto the scaffolds' surface, demonstrating commendable proliferative and migratory capabilities (Figure 2A). By the seventh day, the control group and the SF-Lipo group exhibited a proliferation rate of 3.8 and 3.9 times, respectively, although the difference was statistically insignificant between the two groups (*P *=* *0.2367). Interestingly, the introduction of KGN resulted in a slight enhancement in cell proliferation at the seventh day (Figure 2B). The Live/Dead staining analysis demonstrated that the chondrocytes in each experimental group exhibited high viability, with only a minimal presence of deceased cells, thereby indicating the absence of any noticeable cytotoxic effects exerted by the scaffolds (Figure 2C and D). The wound-healing assays revealed that the introduction of KGN resulted in a 23.7% increase in the migration rate of chondrocytes within the SF-Lipo@KGN group compared to the SF-Lipo group (Figure 2E and F).

### SF-Lipo@KGN scaffold promoted the anabolism of cartilage ECM

**Figure 3.**
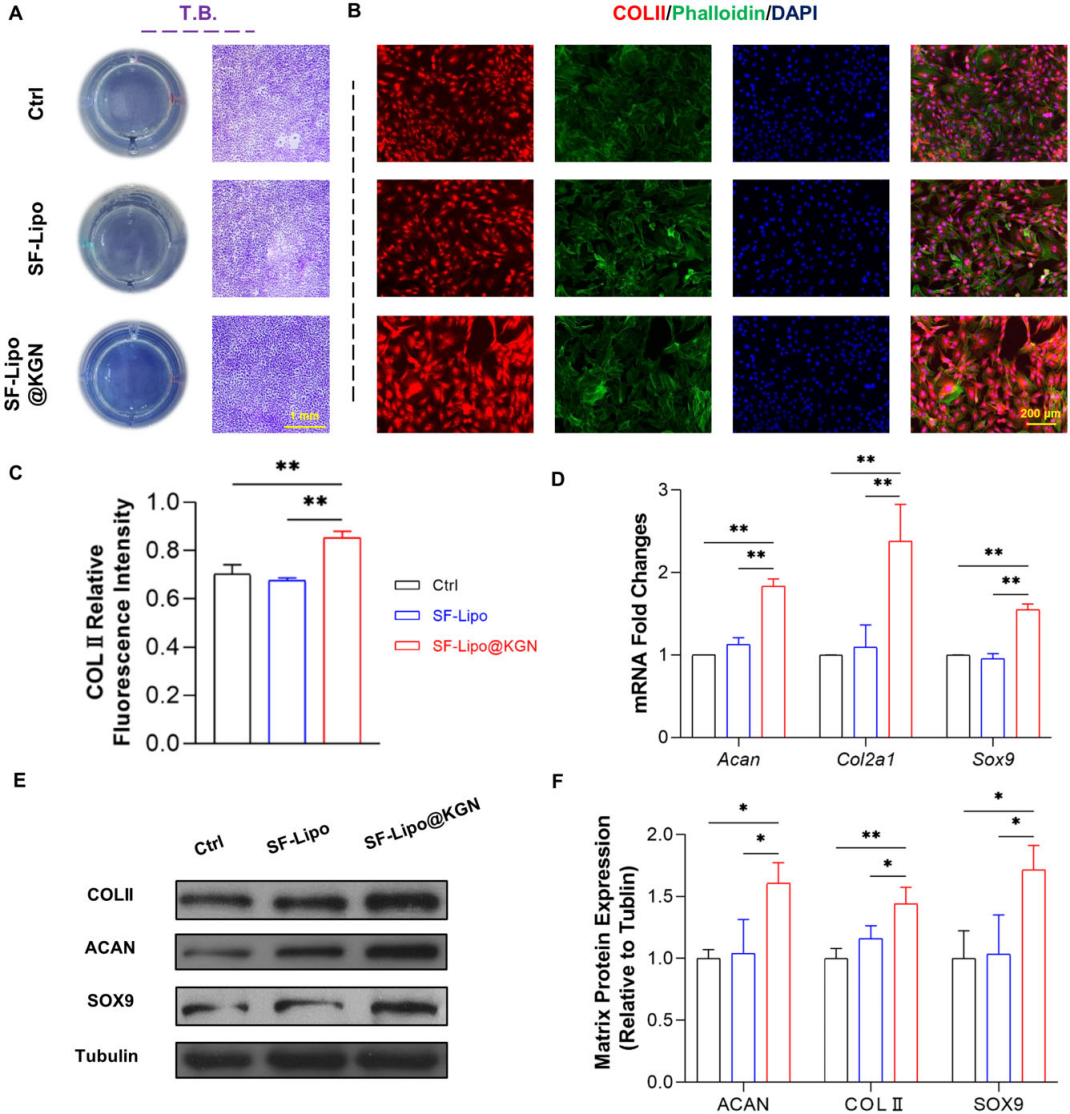
The SF-Lipo@KGN scaffold promotes the synthesis of chondrocyte matrix *in vitro*. (**A**) The deposition of ECM was assessed by toluidine blue staining. Scale bar = 2 mm. (**B, C**) The expression level of cartilage-specific marker COLII was evaluated by immunofluorescence staining. Scale bar = 200 μm. (**D**) The expression of matrix synthesis-related genes (*Col2a1*, *acan*, and *Sox9*) was determined through RT-PCR. (**E, F**) Western blot was used to determine the protein levels of COLII, ACAN, and SOX9. The data are presented as mean ± SD (*n* = 3). *Indicates statistically significant differences where *P *<* *0.05, **where *P *<* *0.01.

To further elucidate the potential influence of the SF-Lipo@KGN scaffolds on cartilage matrix synthesis, the transwell culture system was employed. Toluidine blue staining indicated that the release of KGN from the scaffolds may enhance cartilage anabolic metabolism (Figure 3A). In comparison to the control group, the SF-Lipo@KGN group exhibited a 21.5% increase in the expression of COL II, a crucial constituent of cartilage ECM (Figure 3B and C). Additionally, treatment with SF-Lipo@KGN resulted in an up-regulation of ECM synthesis-related genes, including a 138.0% increase in *Col2a1* expression, an 83.7% increase in *Acan* expression, and a 54.9% increase in *Sox9* expression (Figure 3D). Western blot assays further confirmed that the SF-Lipo@KGN group displayed significantly higher levels of COLII, ACAN, and SOX9, with respective increases of 144.0%, 160.7%, and 171.3% compared to the control group (Figure 3E and F).

### SF-Lipo@KGN scaffolds restored chondrocyte redox homeostasis via activating the NRF2-mediated antioxidant functions

**Figure 4.**
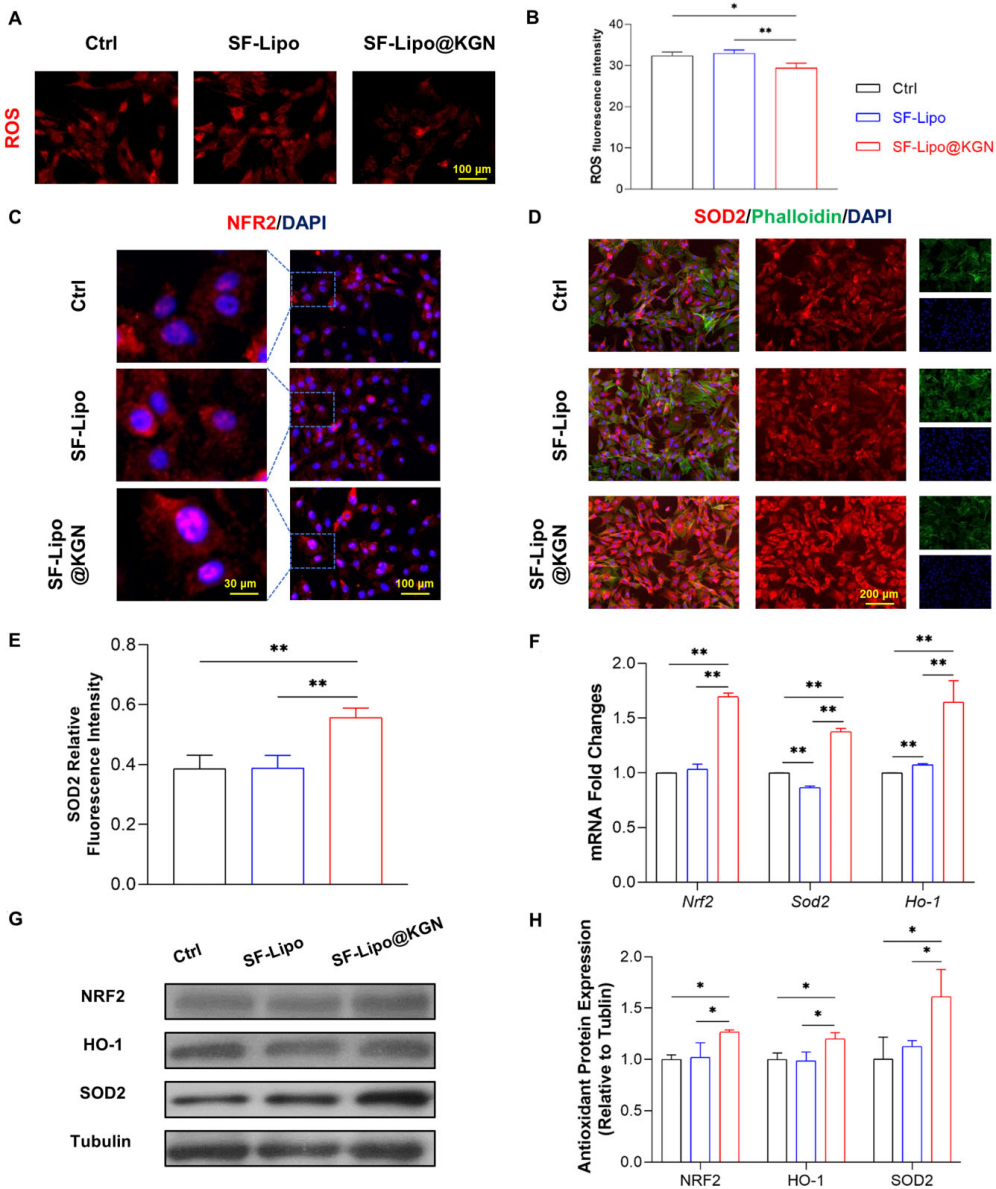
KGN released from scaffolds regulates the antioxidant capacity of chondrocytes. (**A, B**) The ROS level of chondrocytes from various groups was evaluated by immunofluorescence staining. Scale bar = 100 μm. (**C**) The intracellular localization of antioxidant marker transcription factor NRF2 was evaluated by immunofluorescence staining. Scale bar = 30 μm and 100 μm. (**D, E**) Immunofluorescence staining of SOD2 in chondrocytes. Scale bar = 100 μm. (**F**) The expression of antioxidant enzyme genes (*Nrf2*, *Sod2*, and *Ho-1*) was determined through RT-PCR. (**G, H**) Western blotting was used to determine the protein level of NRF2, SOD2, and HO-1. The data are presented as mean ± SD (*n* = 3). *Indicates statistically significant differences where *P *<* *0.05, ** where *P *<* *0.01.

In order to further elucidate the mechanisms underlying the advantageous effects of SF-Lipos@KGN scaffolds on cartilage ECM, our study focused on investigating the NRF2 pathway and the downstream antioxidant enzymes. Following co-culturing of chondrocytes with SF-Lipo@KGN scaffolds, a significant reduction in ROS levels within chondrocytes was observed (Figure 4A and B). Immunofluorescence analysis was conducted to assess the levels and localization of NRF2 and SOD2 in chondrocytes. Notably, the SF-Lipo@KGN scaffolds induced a greater translocation of NRF2 to the nucleus compared to the other two groups (Figure 4C). Furthermore, the expression of SOD2 in the SF-Lipo@KGN group was found to be 44.3% higher than that in the control group (Figure 4D and E). The mRNA expression levels of *Nrf2*, *Ho-1*, and *Sod2* exhibited increases of 69.4%, 64.5%, and 37.4%, respectively (Figure 4F). Additionally, Western blot assays demonstrated that the expression of antioxidant-related proteins increased by 26.7% for NRF2, 20.0% for HO-1, and 61.0% for SOD2 in the SF-Lipo@KGN group (Figure 4G and H).

### Implantation of SF-Lipo@KGN scaffolds boosted cartilage regeneration by modulating endogenous redox microenvironment

**Figure 5.**
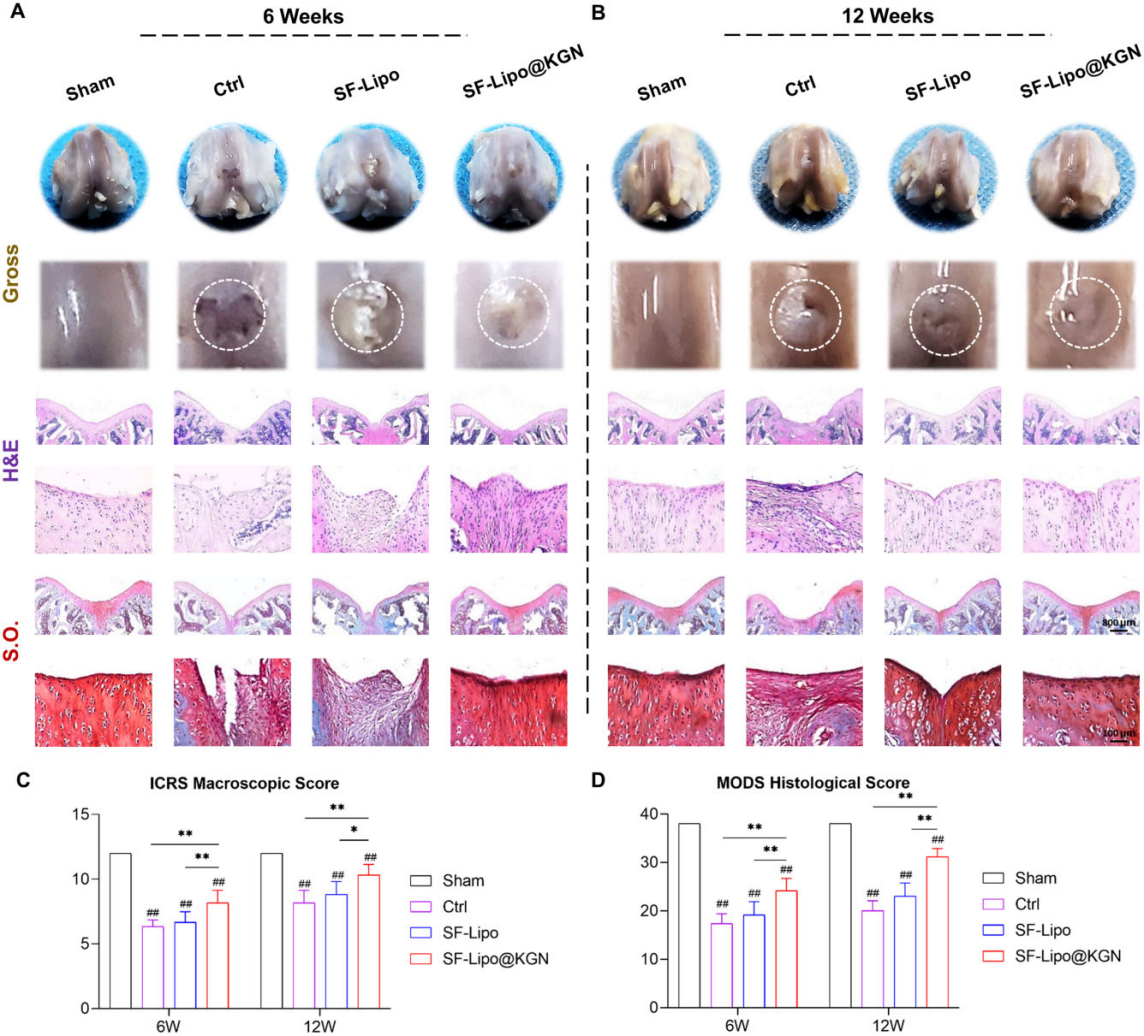
The SF-Lipo@KGN scaffold promotes cartilage defect repair and ECM deposition. (**A, B**) Macroscopic, hematoxylin and eosin (H&E) staining, and safranin O (S.O.) staining representative pictures of cartilage defects at 6 and 12 weeks after the operation. Scale bar = 800 μm and 100 μm. (**C, D**) ICRS macroscopic score and MODS histological score were used to evaluate the degree of cartilage repair. The data are presented as mean ± SD (*n* = 6). *Indicates statistically significant differences where *P *<* *0.05, ** where *P *<* *0.01 between the indicated groups; #where *P *<* *0.05, ##where *P *<* *0.01 versus the sham group.

The composite SF-Lipo@KGN scaffolds were implanted into rat full-thickness cartilage defects to assess their potential regenerative effects. At 6 weeks post-surgery, H&E and S.O staining demonstrated the presence of newly formed tissue within the cartilage defects in both the control and SF-Lipo groups. However, the SF-Lipo@KGN group exhibited the regeneration of thicker tissue resembling hyaline cartilage (Figure 5A). At 12 weeks post-surgery, both the SF-Lipo@KGN and SF-Lipo groups displayed satisfactory tissue filling. Nevertheless, the SF-Lipo@KGN group exhibited superior deposition of cartilage matrix within the defect (Figure 5B). After 6 weeks of surgery, the SF-Lipo@KGN group exhibited a 28.9% increase in ICRS macroscopic scores and a 39.4% increase in MODS histological scores compared to the control group (Figure 5C). Similarly, after 12 weeks of surgery, SF-Lipo@KGN demonstrated an expected enhancement in cartilage repair potential, as evidenced by a 26.5% increase in ICRS macroscopic scores and a 55.8% increase in MODS histological scores, compared with the control group (Figure 5D).

**Figure 6.**
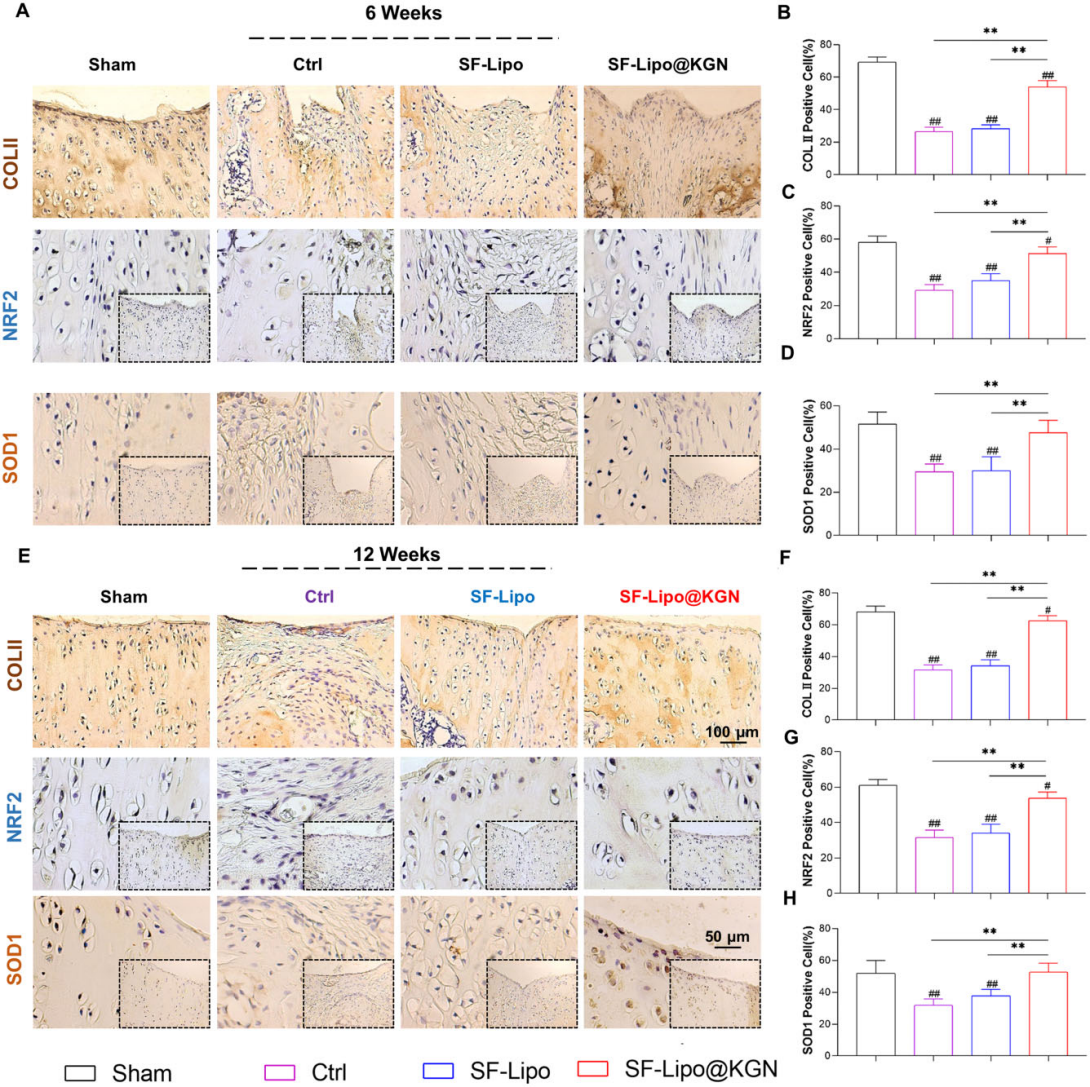
The SF-Lipo@KGN scaffold promotes the expression of matrix protein and antioxidant protein in cartilage tissue. (**A**) Representative IHC images of cartilage defects stained with COLII, NRF2 and SOD1 at 6 weeks after surgery. Scale bar = 100 μm and 50 μm. (**B–D**) Quantifying the percentage of COLII-positive, NRF2-positive, and SOD2-positive cells in the articular cartilage at 6 weeks after surgery. (**E**) Representative IHC images of cartilage defects stained with COLII, NRF2 and SOD1 at 12 weeks after surgery. Scale bar = 100 μm and 50 μm. (**F–H**) Quantifying the percentage of COLII-positive, NRF2-positive, and SOD1-positive cells in the articular cartilage at 12 weeks after surgery. The data are presented as mean ± SD (*n* = 6). *Indicates statistically significant differences where *P *<* *0.05, ** where *P *<* *0.01 between the indicated groups; # where *P *<* *0.05, ## where *P *<* *0.01 versus the sham group.

Consistently, the therapeutic effects were confirmed through immunohistochemical staining. Immunostaining results revealed that the implantation of SF-Lipo@KGN yielded a higher production of COL II compared to the control and SF-Lipo groups. After 6 and 12 weeks post-surgery, the regenerative tissue in the SF-Lipo@KGN group closely resembled natural cartilage observed in the Sham group, while the control group exhibited a greater presence of fibrous tissue (Figure 6A and E). Furthermore, the level of COL II in chondrocytes of the SF-Lipo@KGN group was significantly elevated by 103.3% and 99.0% at 6 and 12 weeks, respectively, in comparison to the control group (Figure 6B and F). Moreover, the immunostaining analysis of NRF2 demonstrated a significant augmentation in the expression of NRF2 in the SF-Lipo@KGN group (Figure 6C and G). In comparison to the control group, the SF-Lipo@KGN group exhibited a notable increase of 61.1% at 6 weeks post-surgery and 65.3% at 12 weeks post-surgery in the percentages of SOD1-positive cells. These results strongly indicate that implantation of SF-Lipo@KGN scaffolds promoted a remarkable synthesis of hyaline cartilage ECM and enhanced the antioxidant functions in the defect site.

## Discussion

The present study provides evidence that the abnormal metabolism of ECM and oxidative stress in cartilage play a significant role in the progression of osteoarthritis [32]. Our findings demonstrate that KGN, a small hydrophobic molecule, has the potential to protect articular cartilage from oxidative injury. Specifically, our results indicate that KGN-loaded scaffolds effectively reduce intracellular ROS, increase the expression of the antioxidant enzyme HO-1 at both mRNA and protein levels, and enhance the synthesis of cartilage ECM. Furthermore, we elucidate the underlying molecular mechanism by which KGN exerts its antioxidant effects, involving the NRF2 signaling pathway. The disintegration of filamin A (FLNA) and the core-binding factor β (CBFβ) has revealed the potential of KGN for cartilage regeneration, as it activates chondrogenic differentiation through the regulation of the runt-related transcription factor 1 (RUNX1) [15]. The Smad signaling pathway plays a crucial role in the regulation of chondrogenesis and cartilage homeostasis, being activated by the binding of TGF-β to its receptor, leading to the recruitment and phosphorylation of Smad2 and Smad3, followed by their binding to Smad4. The complex undergoes translocation to the nucleus, where it exerts regulatory control over the expression of target genes associated with chondrogenesis and ECM synthesis [33]. Our observations consistently revealed that KGN-loaded scaffolds facilitated the synthesis of cartilage matrix components, such as COLII and aggrecan. A prior investigation substantiated that KGN suppressed the expression of collagen X while concurrently up-regulating proteins involved in matrix synthesis, thereby indicating that KGN fosters the formation of cartilage matrix while impeding chondrocyte hypertrophy and endochondral ossification [34]. In addition, Liu *et al*. discovered that KGN has the potential to enhance the expression and release of lubricin protein, potentially by activating the c-Myc and ADAMTS5 pathways [35]. Consequently, KGN could facilitate the cartilage repair process by modulating chondrogenic and antioxidant signaling pathways.

The impact of oxidative stress at the site of injury on cartilage repair outcomes is noteworthy, as excessive ROS can induce chondrocyte apoptosis, disrupt cartilage matrix metabolism, and trigger the release of pro-inflammatory cytokines [36]. The results of our investigation demonstrate that SF-Lipo@KGN scaffolds effectively mitigate local oxidative stress by decreasing ROS levels in chondrocytes. A prior study conducted in our laboratory proposed that the influence of KGN on intracellular antioxidant mechanisms involves the suppression of microRNA-146a to modulate post-transcriptional regulation of antioxidant proteins [19]. In the present study, we have discovered that KGN enhances intracellular antioxidant functions through the activation of the NRF2 pathway, which governs the expression and activity of diverse antioxidant enzymes such as SODs, catalase, GPx, and HO-1. Specific deletion of *Nrf2* led to significant degradation of cartilage in a mouse model of arthritis, while the introduction of NRF2 with trichostatin A (TSA) effectively reversed the detrimental effects [37]. Consequently, the NRF2/HO-1 signaling pathway exhibits a protective role in safeguarding cartilage against oxidative stress and inflammatory harm induced by diverse stressors [38]. Zhou *et al*. discovered that the activation of the NRF2/HO-1 pathway through melatonin, an endogenous hormone associated with circadian rhythm, shielded chondrocytes from damage caused by oxidative stress by upregulating the expression of antioxidant enzymes [39]. Our research has provided evidence demonstrating that KGN not only increased the expression of NRF2 but also facilitated its translocation to the nucleus, thereby enhancing the transcription of downstream antioxidant enzymes, such as HO-1 and SOD2. These findings substantiate that the release of KGN from SF-Lipo@KGN scaffolds enhances the anabolism of the cartilage matrix by restoring redox homeostasis in chondrocytes.

The controlled release of drugs targeted specifically to the affected area is a crucial aspect of cartilage regeneration. Previous studies have demonstrated the effectiveness of intra-articular injection of KGN in alleviating osteoarthritis [40]. However, in the context of localized cartilage defects, the administration of KGN may lead to unintended adverse effects. A study conducted by Yuan *et al*. revealed that the introduction of KGN-loaded alginate beads into the Achilles tendons of rats resulted in the emergence of ectopic chondrocytes and the formation of cartilage matrix, thereby implying the potential induction of tendinosis by KGN [41]. Furthermore, the limited water solubility of KGN hinders its metabolic pathway within organisms, thereby posing a significant obstacle to its effective utilization. This obstacle may consequently lead to the accumulation of the drug, crystallization, and an increased vulnerability to toxic side effects. To address these concerns, Yang *et al*. utilized liposome encapsulation as a means to mitigate potential challenges associated with hydrophobic molecules. They devised an innovative approach involving GelMA microspheres loaded with liposomes containing KGN, and successfully demonstrated the efficacy of these composite microspheres in lubricating the joint cavity and mitigating the progression of osteoarthritis through localized release of KGN [42]. However, the objective of this study was to enhance cartilage regeneration by incorporating SF scaffolds and liposome-encapsulated KGN. In contrast to GelMA, SF-Lipo@KGN scaffolds demonstrated enhanced mechanical properties (compression modulus) and a prolonged degradation period, despite displaying inferior lubrication performance [43]. This renders SF-Lipo@KGN scaffolds a viable alternative for addressing cartilage defects.

The sustained release of KGN from SF scaffolds aligns with the gradual nature of cartilage repair. Additionally, the positive charge on the surface of cationic liposomes facilitates binding to chondrocyte surfaces through electrostatic interactions. Water molecules have the ability to attach themselves to the phosphocholine headgroups of liposomes, resulting in the formation of hydration layers that can be continuously replenished [44]. This phenomenon leads to a notable lubricating effect. Furthermore, SF-Lipo@KGN scaffolds demonstrated a degradation period lasting more than 8 weeks and were able to provide prolonged mechanical support during the cartilage repair process. The scaffolds were produced using a freezing technique, devoid of supplementary cross-linking agents, which effectively reduces the potential cytotoxicity risks. This method promotes the migration of cartilage cells and facilitates the deposition of matrix, thereby mimicking the mechanical characteristics of native hyaline cartilage [45]. Consequently, the SF-Lipo@KGN scaffolds enable localized release of KGN at the site of injury, while also satisfying the necessary material properties for cartilage defect restoration.

There are several limitations that should be acknowledged in relation to this study. Firstly, it is important to recognize the undeniable influence of the inflammatory environment in damaged tissue on the destiny of chondrocytes. Our investigation primarily focused on examining the impact of KGN on the antioxidant functions and matrix metabolism of chondrocytes under normal culture conditions. However, the protective effect of KGN against the inflammatory microenvironment remains uncertain. Consequently, it is imperative to further explore the effect of KGN on the antioxidant system in chondrocytes when exposed to proinflammatory cytokines. Secondly, the release of KGN from the SF scaffolds resulted in inevitable contact with various tissues within the joint cavity, such as the synovium, subchondral bone, and infrapatellar fat pad. Given the potential risks associated with KGN, forthcoming investigations will prioritize examining the effects of KGN on the tissues within the joint cavity. Lastly, it is important to note that our current research has solely focused on the early repair of uncomplicated cartilage defects. In future studies, double-layer scaffolds will be developed to independently address the repair of both cartilage and subchondral bone.

## Conclusion

We employed a facile methodology to develop composite scaffolds through incorporation of SF scaffolds and liposome-encapsuled KGN. The resulting porous constructs of the SF-Lipo@KGN scaffolds exhibit exceptional connectivity and mechanical properties, potentially enhancing cell adhesion, migration, and nutrient supply. Notably, the SF-Lipo@KGN scaffolds demonstrated advantageous pro-anabolic effects by stimulating the synthesis of cartilage matrix and activating antioxidant enzymes to restore redox homeostasis. In conclusion, our investigation presents a biomaterial with redox-modulatory properties that holds promise as a candidate for cartilage tissue engineering.
